# A Fatal Case of *Candida auris* and *Candida tropicalis* Candidemia in Neutropenic Patient

**DOI:** 10.1007/s11046-018-0244-y

**Published:** 2018-01-30

**Authors:** Ratna Mohd Tap, Teck Choon Lim, Nur Amalina Kamarudin, Stephanie Jane Ginsapu, Mohd Fuat Abd Razak, Norazah Ahmad, Fairuz Amran

**Affiliations:** 1 Bacteriology Unit, Infectious Diseases Research Centre, Institute for Medical Research, Jalan Pahang, 50588 Kuala Lumpur, Malaysia; 2 Haematology Unit, Hospital Raja Permaisuri Bainun, Jalan Hospital, 30990 Ipoh, Perak Malaysia

**Keywords:** Mixed candidemia, Multidrug resistant, *Candida auris*

## Abstract

We report a fatal case of *Candida auris* that was involved in mixed candidemia with *Candida tropicalis*, isolated from the blood of a neutropenic patient. Identification of both isolates was confirmed by amplification and sequencing of internal transcribed spacer and D1/D2 domain of large subunit in rRNA gene. Antifungal susceptibility test by E-test method revealed that *C. auris* was resistant to amphotericin B, anidulafungin, caspofungin, fluconazole, itraconazole and voriconazole. On the other hand, *C. tropicalis* was sensitive to all antifungal tested. The use of chromogenic agar as isolation media is vital in detecting mixed candidemia.

## Introduction

Earlier before the introduction of chromogenic agar and molecular methods, candidemia has been generally considered to be an infection caused by one *Candida* species, due to the limitation of conventional microbiological techniques which were not able to distinguish more than one species of yeast in patient’s samples. However, for the past two decades, with the growing numbers of immunocompromised and the improvement of diagnostic methods, the probability of the clinician encountered mixed infection of candidemia is increasing [1, 2].

The incidence of mixed candidemia (MC) is relatively low, ranging from 2 to 9.3% of total candidemia [3–5]. *Candida albicans* is the common species involved in MC, and the most common combination was *C. albicans* + *C. glabrata* [6] and *C. albicans* + *C. parapsilosis* [2]. Other species including *C. tropicalis, C. krusei, C. dubliniensis* and *C. glabrata* also had been reported to cause MC [2, 6]. However, uncommon *Candida* species, particularly *C. auris,* has never been reported in MC cases. *C. auris* is an emerging, multidrug-resistant yeast that causes invasive infections and is transmitted in health care settings. Since it was first described in 2009, cases of *C. auris* candidemia had been reported in Asia, Europe, the Middle East and the USA [7–10]. In fact, there have been discrete outbreaks globally, especially in the UK and the USA [11]. This is the first report on isolation of *C. auris* from the blood of neutropenic patient, involved in a MC with *C. tropicalis*.

## The Patient

A 63-year-old retired construction worker with no significant medical history was presented to Kampar Hospital with generalized colicky abdominal pain associated with loose stool of 1-week duration and no bleeding tendency. He was afebrile but complained loss of appetite and weight for the past 1 month. On examination, the patient was noted to be pale with fever of 38 °C. No abnormality and no lymphadenopathy were detected during abdominal examination. Other respiratory, cardiovascular and muscular and neurology systems were unremarkable.

Initial complete blood count on the day of admission revealed pancytopenia with low reticulocyte response. Full blood picture examination revealed leukopenia and dysplastic neutrophils. He was referred to the state’s hospital for further investigation and management with the working diagnosis of febrile neutropenia to rule out myelodysplastic syndrome.

He was started on intravenous (IV) tazocin 4.5 g four times a day and IV gentamicin 120 mg daily. Septic work out (blood and urine culture) that was performed during day 1 and day 4 admissions was negative for any micro-organism. However, gram stain from the blood culture which was taken on day 9 demonstrated yeast-like cells. Subsequently, a dosage of 400 mg/day once a day of IV fluconazole was started following the positive finding.

Despite antifungal treatment given, the clinical condition of the patient remained febrile throughout his hospital stay. On day 15, IV imipenem 500 mg was started due to persistent temperature of 40 °C. On the next day, his Glasgow Coma Scale suddenly dropped from full score to M4 V2 E2, and he was subsequently intubated. Computed tomography finding of the brain revealed left parietal region bleed with midline shift. Nevertheless, neurosurgical team was unable to proceed with surgical intervention as his platelet count was still low despite the transfusion given. Patient’s clinical condition deteriorated further, and he succumbed to the illness 2 days later.

## Mycological Identification

**Fig. 1:**
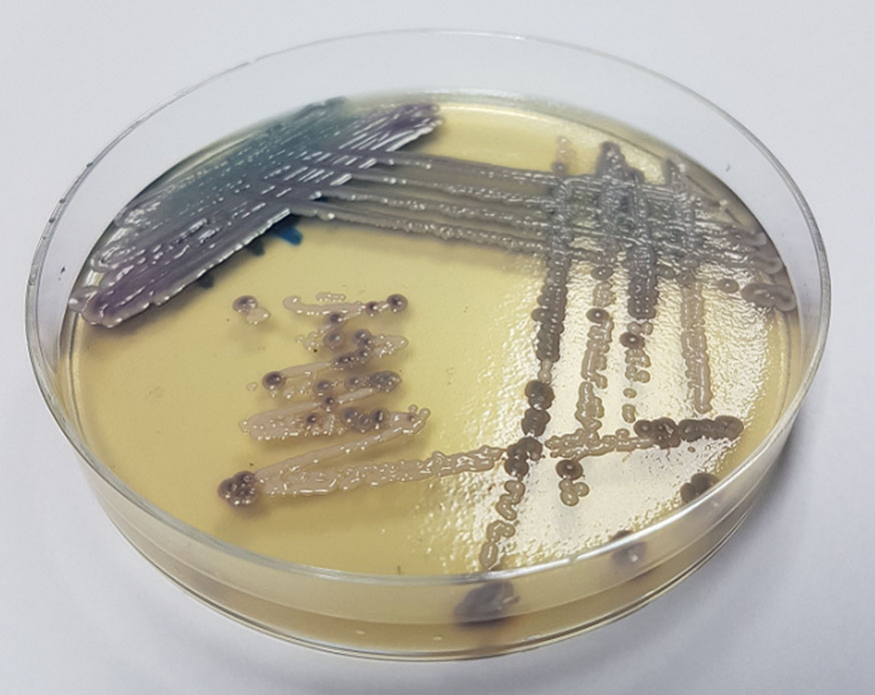
Mix growth of two yeast-like colonies produced distinguish colour on CHROMagar Candida plate after 48-h incubation

**Fig. 2:**
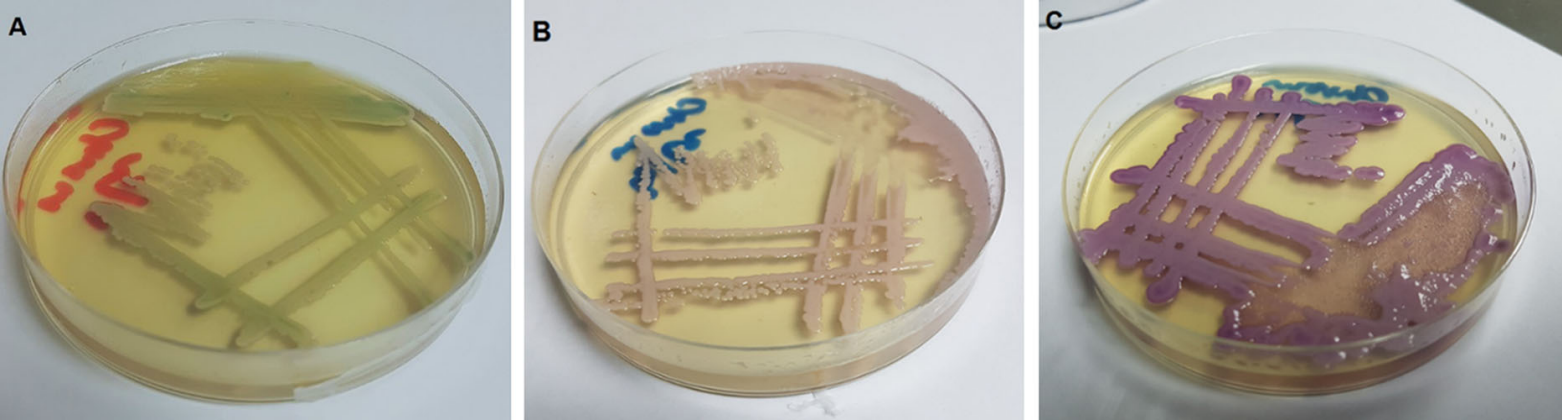
Pure culture of both isolates on CHROMagar Candida. After 48-h incubation at 30 °C, UZ1446 produces very weak blue-green colour (**a**), while UZ1447 produces pink colour (**b**). Nonetheless, after prolong incubation (120 h), the colour of UZ1447 colonies became violet (**c**), while UZ1446 remained the same

**Fig. 3:**
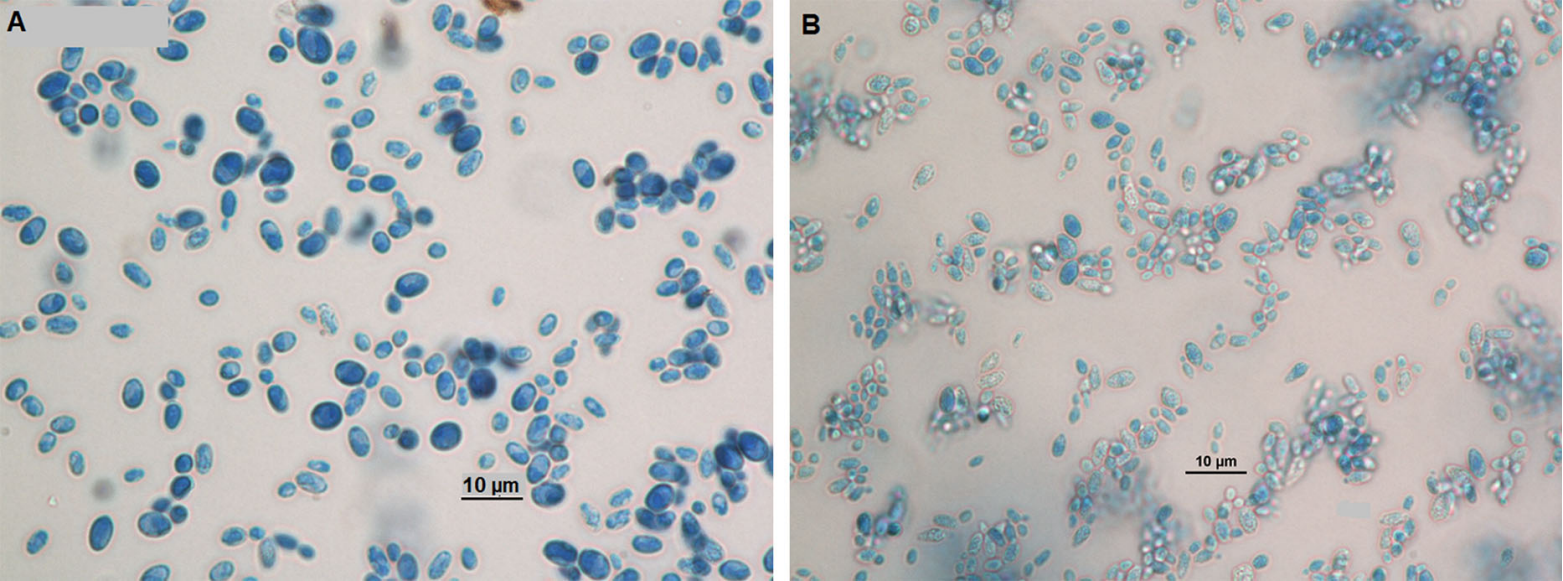
Lactophenol cotton blue staining from the 48-h-old culture on SDA exhibited ovoid and larger blastoconidia of UZ1446, later was identified as *C. tropicalis* (**a)**; ellipsoidal and smaller blastoconidia of UZ1447, later was identified as *C. auris* (**b**) at magnification of ×100

**Fig. 4:**
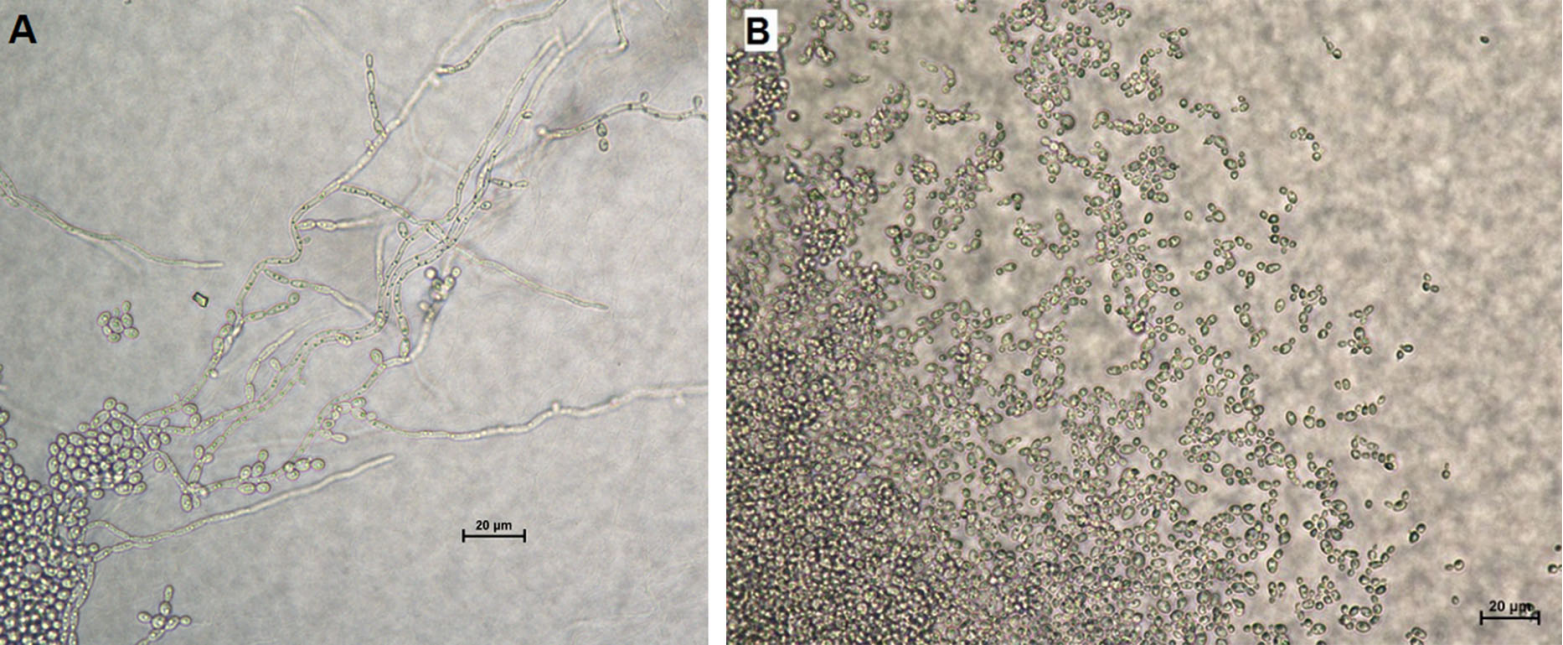
Slide culture on corn meal agar of UZ1446 (*C. tropicalis)* demonstrating abundance, long and branched pseudohyphae with single or in cluster blastoconidia on the pseudohyphae and (**a**) the absence of pseudohyphae for UZ1447 (*C. auris*) and (**b**) at magnification of ×40

Gram stain performed on the positive blood culture revealed yeast-like cells. The blood was subcultured on a Sabouraud dextrose agar (SDA) plate and incubated at 35 °C. After 48 h of incubation, few yeast-like colonies were observed on SDA plate. The plate was sent to Mycology Laboratory in Institute for Medical Research (IMR) for further identification. Upon receival at the laboratory, the colonies were subcultured onto a new SDA plate and CHROMagar Candida (Becton Dickinson, Heidelberg, Germany) plate, incubated at 30 °C. After 48 h of incubation, cream-coloured, smooth, glabrous and yeast-like colonies were observed on SDA plate; however, on the CHROMagar Candida plate, on the heavy inoculation, there were two different colours of yeast-like colonies seen, i.e. blue and dark purple colonies (Fig. 1). The individual colony of two different colour colonies was subcultured onto new CHROMagar Candida and SDA plates for further tests. The blue and dark purple colonies were given strains name as UZ1446 and UZ1447, respectively. After 48 h incubation at 30 °C, both isolates had similar appearance on SDA plates, i.e. smooth, shiny and cream-coloured colonies. However, on new CHROMagar Candida plates, both isolates displayed distinguish colour (Fig. 2). Isolate UZ1447 produced violet colour colonies on CHROMagar Candida plate after prolonged incubation at 120 h (Fig. 2c). Microscopically, blastoconidia of isolate UZ1446 were ovoid with the size (1.9–3.4) × (2.7–4.8) µm; UZ1446 was ellipsoidal with the size (2.5–4.2) × (4.5–5.6) µm (Fig. 3). Budding was also observed in both isolates. Slide culture on corn meal agar (supplemented with 2% Tween-80) was performed for both isolates. After 48 h incubation at 30 °C, isolate UZ1446 produced abundance, long and branched pseudohyphae with single or in cluster blastoconidia on the pseudohyphae. However, isolate UZ1447 did not produce pseudohyphae on the slide culture (Fig. 4).

Carbohydrate assimilation test for both isolates was carried out using commercial systems, API 20C and Vitek 2 system (bioMe´rieux, Marcy l’Etoile, France). On both systems, isolate UZ1446 was identified as *C. tropicalis* with high probability. However, for isolate UZ1447, it was identified as *Rhodotorula glutinis* with 99.3% probability on API 20C system and as C*. haemulonii* with 97% probability on Vitek 2 system (Table 1).

**Table 1:** Identification of isolates UZ1446 and UZ1447 using API 20C, Vitek 2 and BLAST search of ITS and LSU regions

Isolate identification system	UZ1446	UZ1447
API 20C (%)	*C. tropicalis* (99.8)	*Rhodotorula glutinis* (99.3)
Vitek 2 (%)	*C. tropicalis* (99)	*C. haemulonii* (97)
ITS (%), GenBank accession number	*C. tropicalis* (100) Not submitted	*C. auris* (98) KP326583
LSU (%), GenBank accession number	*C. tropicalis* (100) Not submitted	*C. auris* (100) KU321688

## Molecular Identification

DNA extractions, amplifications by polymerase chain reaction (PCR), PCR product purification and sequencing methods were performed as previously described [12]. The internal transcribed spacer (ITS) and the D1/D2 domain of the large subunit (LSU) in rRNA gene regions were amplified using universal primers ITS5/ITS4 [13] and NL1/NL4 [14]. NCBI BLAST search (http://blast.ncbi.nlm.nih.gov/) was performed for the ITS and LSU sequences from both isolates. However, only sequences of isolate UZ1447 had been submitted to the GenBank (Table 1).

## In Vitro Antifungal Susceptibility Testing (AST)

In vitro AST was carried out using the E-test method, performed according to the manufacturer’s protocols (AB Biodisk, Solna, Sweden). The antifungals tested were amphotericin B, caspofungin, anidulafungin, fluconazole, itraconazole and voriconazole. The minimum inhibitory concentration (MIC) of the antifungals was determined after 48 h incubation and interpreted following the Clinical and Laboratory Standards Institute (CLSI), 2012 guidelines [15]. In vitro susceptibility pattern showed that isolate UZ1446 was sensitive to all antifungals tested. On the other hand, isolate UZ1447 was resistant to all antifungal tested, i.e. amphotericin B, anidulafungin, caspofungin, fluconazole, itraconazole and voriconazole, with no zone was formed on the AST plate for caspofungin, fluconazole and voriconazole (Table 2).

**Table 2:** MICs and susceptibility interpretations of the isolates against antifungals

Antifungal	MIC (µg ml^−1^)	
	UZ1446 (*C. tropicalis*)	UZ1447 (*C. auris*)
Amphotericin B	0.002 (S)	3.000 (R)
Caspofungin	0.002 (S)	> 32.000 (R)
Anidulafungin	< 0.002 (S)	0.750 (R)
Fluconazole	0.750 (S)	> 256.000 (R)
Itraconazole	0.125 (S)	4.000 (R)
Voriconazole	0.047 (S)	> 32.000 (R)

*S* sensitive, *R* resistant

## Discussion

This study reported a fatal case of *C. auris*, involved in mixed candidemia with *C. tropicalis*, isolated from neutropenia patient. The identification of both isolates was confirmed based on PCR sequencing of ITS region and D1/D2 domain in LSU region of the rRNA genes. The *C. auris* exhibited resistance to amphotericin B, anidulafungin, caspofungin, fluconazole, itraconazole and voriconazole.

On SDA, both isolates produced almost the same characteristics macro- and microscopically. Fortunately, chromogenic media like CHROMagar Candida was able to differentiate two types of colonies. The media is very useful to reveal of MC, in which if it is not been included in isolation media, MC can remain undetected by routine isolation methods. Few studies had shown the usefulness of chromogenic media in detecting mixed candidemia [2, 6, 16–19]. In their studies, the incidence of MC ranged from 2.8 to 5.2%, with the most common species involved were *C. albicans, C. parapsilosis, C. tropicalis* and *C. glabrata.* Few *Candida* species including *C. famata, C. krusei, C. lusitaniae, C. guilliermondii* and *C. dubliniensis* were also reported but rarely caused MC. To the best of our knowledge, *C. auris* has never been reported to cause MC, thus this study is the first to report on MC involving *C. auris.*

Uncommon *Candida* species was difficult to identify using conventional phenotypic methods. The database limitation of commercial identification system may lead incorrect identification of these species. Thus, PCR sequencing of the ITS and D1/D2 domain in LSU regions is one of the reliable methods for uncommon *Candida* species identification [20, 21]. Besides that, MALDI-TOF that uses protein for identification was able to identify *C. auris* [22]. Thus, in laboratories that rely on commercial identification systems, some important species like *C. auris* was under reported. The incorrect identification of multidrug resistance species will lead to the inappropriate antifungal treatment to the patient especially to those who are immunocompromised. Awareness on this issue should be emphasized among the diagnostic laboratories, so that the precautions can take place to avoid transmission of multidrug resistance *C. auris* in health care settings.

In this study, in vitro AST of *C. tropicalis* and *C. auris* revealed different susceptibility patterns. While *C. tropicalis* was all sensitive, *C. auris* was resistance to six antifungal drugs tested. The finding explained why clinical condition of the patient remained febrile, even though he was treated with fluconazole. Nevertheless, the patient died before the antifungal susceptibility results were available. Neutropenia condition, prolonged hospital stay, the use of broad-spectrum antibiotics and the presence of central venous catheters had been identified as risk factors which exposed immunocompromised patient to candidemia [23, 24]. After the first isolation of *C. auris*, to date we have not received other *C. auris* isolates from the same or any other hospital in Malaysia. Under-reported cases might be the reason why this situation happened.

## Conclusion

The use of chromogenic agar as isolation media is vital in detecting mixed candidemia. Due to the limitation of commercial system like API 20C and Vitek 2, PCR sequencing method is foremost in identifying uncommon yeast species. With resistance to the main antifungal groups i.e. azoles, polyene and echinocandin, the treatment for *C. auris* infection has now become very challenging.
